# Inexpensive and Highly Reproducible Cloud-Based Variant Calling of 2,535 Human Genomes

**DOI:** 10.1371/journal.pone.0129277

**Published:** 2015-06-25

**Authors:** Suyash S. Shringarpure, Andrew Carroll, Francisco M. De La Vega, Carlos D. Bustamante

**Affiliations:** 1 Department of Genetics, Stanford University, Stanford, California 94305, USA; 2 DNAnexus, Mountain View, California 94040, USA; 3 Real Time Genomics, Inc. San Bruno, California 94066, USA; Technische Universität Dresden, Medical Faculty, GERMANY

## Abstract

Population scale sequencing of whole human genomes is becoming economically feasible; however, data management and analysis remains a formidable challenge for many research groups. Large sequencing studies, like the 1000 Genomes Project, have improved our understanding of human demography and the effect of rare genetic variation in disease. Variant calling on datasets of hundreds or thousands of genomes is time-consuming, expensive, and not easily reproducible given the myriad components of a variant calling pipeline. Here, we describe a cloud-based pipeline for joint variant calling in large samples using the Real Time Genomics population caller. We deployed the population caller on the Amazon cloud with the DNAnexus platform in order to achieve low-cost variant calling. Using our pipeline, we were able to identify 68.3 million variants in 2,535 samples from Phase 3 of the 1000 Genomes Project. By performing the variant calling in a parallel manner, the data was processed within 5 days at a compute cost of $7.33 per sample (a total cost of $18,590 for completed jobs and $21,805 for all jobs). Analysis of cost dependence and running time on the data size suggests that, given near linear scalability, cloud computing can be a cheap and efficient platform for analyzing even larger sequencing studies in the future.

## Introduction

Whole-genome sequencing of population cohorts will be critical for understanding the contribution of rare genetic variation to health and disease and the demographic history of our species. With falling costs, it is now possible to sequence genomes of many individuals for association studies and other genomic analyses. Using low-coverage whole-genome sequencing of many individuals from diverse human populations, the 1000 Genomes Project has characterized common variation and a considerable proportion of the rare variation present in human genomes [1, 2]. Variant calling on large genomic datasets is expensive in terms of computation time and storage and rarely reproducible, given the myriad components of informatics pipelines. Furthermore, while population-based calling has many advantages for improved genotype quality and variant detection, many investigators opt for “single sample calling” for convenience and cost. Using cloud computing, such large computation-intensive tasks can be performed efficiently and reproducibly.

The primary advantage of cloud computing is that it enables the user to request computing and storage resources on-demand without having to own and maintain a computer or cluster of computers required for large data analysis tasks. As a result, pipelines that are run in the cloud can be easily scaled to analyze massive datasets. Another advantage of cloud-based data analysis pipelines is they enable users to effectively utilize any available parallelism in the analysis by requesting many computers simultaneously.

A number of cloud-based pipelines are available for analyses of sequencing data: StormSeq [3] and CloudBurst [4] for read mapping; Crossbow [5] and Mercury [6] for mapping and variant calling etc. A significant limitation of these pipelines is that they can only identify variants within a single sample. While this approach has high power for detecting variants in high-coverage sequencing, it performs worse than multisample calling when applied to low-coverage sequencing data [1]. Huang et al. [7] have deployed the multisample SNPTools pipeline to the Amazon cloud and demonstrated its use for the same variant calling task we report. We compare the two approaches in more detail in the Discussion section.

We have developed a scalable cloud-based pipeline for joint variant calling in large samples. It uses the multisample caller from Real Time Genomics [8] deployed to the Amazon cloud via the DNAnexus platform. Our method has three main advantages:

1. Our pipeline is based in the Amazon cloud and is, thus, not constrained by local compute or storage limitations. Using the Amazon cloud, it can be easily scaled to bigger datasets.
2. It can be parallelized over data split by chromosomes and populations (Methods). Users can change the amount of parallelism according to computation time and cost constraints.
3. Management of Amazon cloud computing resources is handled by DNAnexus to make the pipeline user-friendly.

**Fig 1:**
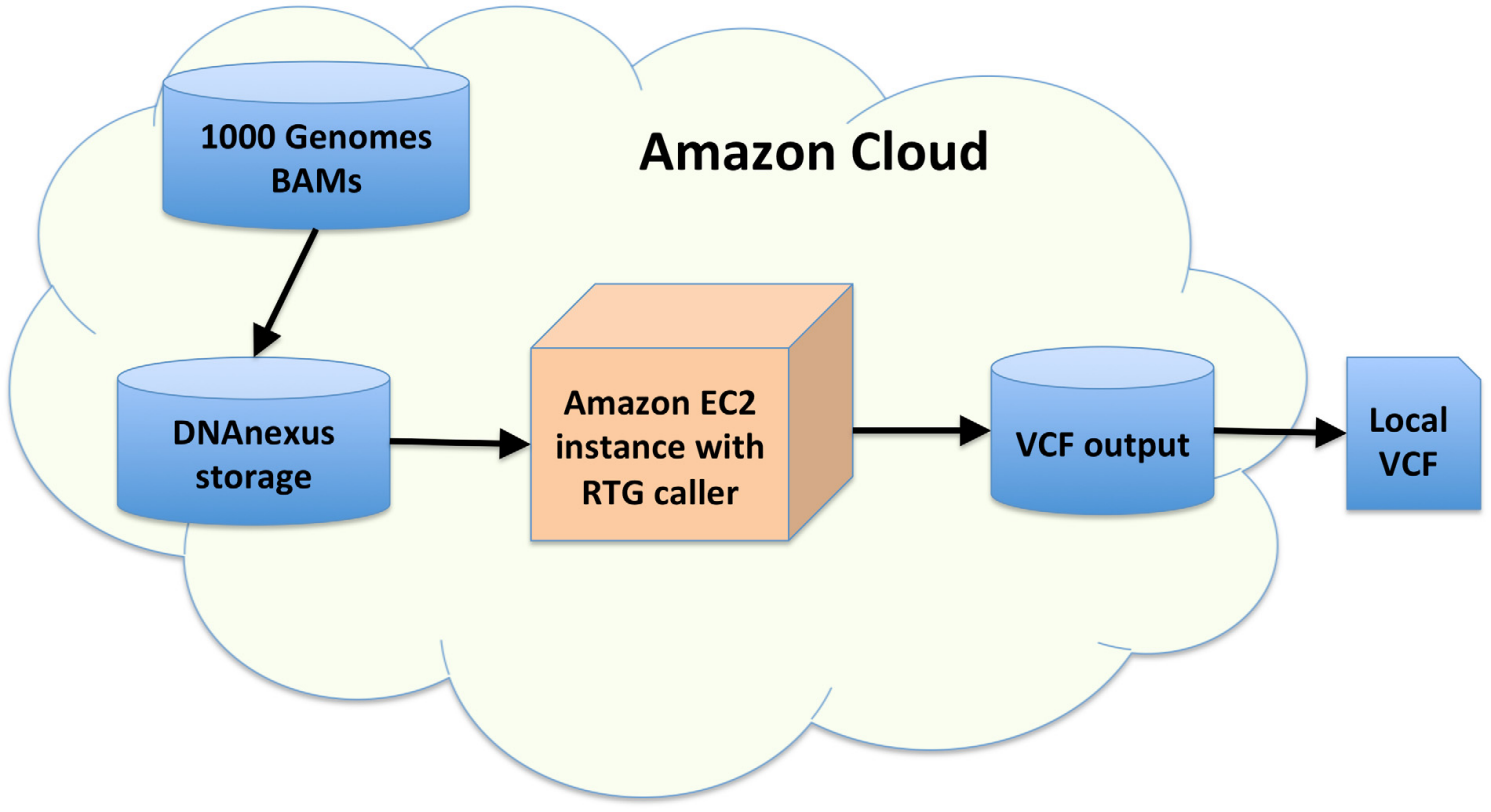
Variant calling pipeline. Diagram of variant calling pipeline. Blue indicates storage and orange indicates computing.

Fig 1 shows a representation of the pipeline. To maximize parallelism, variant calling was performed separately for each chromosome and population in 572 parallel jobs. For a given chromosome and population, alignment files (BAMs) were transferred from 1000 Genomes Amazon cloud storage to DNAnexus Amazon cloud storage. We uploaded the RTG population caller to DNAnexus and allocated Amazon Elastic Compute Cloud (EC2) computing instances capable of running the software. On these instances, the downloaded BAMs were processed using the RTG population caller. The output VCF files were stored in the DNAnexus storage and later downloaded to local storage. A detailed description of the components of the pipeline can be found in the Methods section.

We used our pipeline to identify variants in 2,535 individuals from Phase 3 of the 1000 Genomes Project. We found 68.3 million variants across the samples in 5 days at a total cost of $18,590, equivalent to a per-sample cost of $7.33. We analyzed the computational performance and cost of our pipeline with respect to the size of the alignment data and showed that it offers a feasible means of jointly calling variants in even larger samples at a reasonable cost and running time. It is important to note that the samples from the 1000 Genomes Project we use here were sequenced to low-coverage and the key intent was variant discovery using a multi-sample framework. Additional modifications of the pipeline are likely required for clinical use where the key intent is maximizing the quality of individual genotype calls and ensuring reproducibility of the clinical genomics workflow (often in a CLIA/CAP setting).

## Results

We used our pipeline to identify variants in 2,535 samples from Phase 3 of the 1000 Genomes Project. Below, we describe the variant calls and the computational details of the variant calling.

### Variant callset

We found 61,995,965 SNPs and 6,263,878 indels, a total of 68.3 million variants, across the 2,535 samples. Using monomorphic sites from the Omni array, we evaluated the false positive rate for our variant calling to be 2.5%, consistent with expectations for the 1000 Genomes Project. We found that the transition-transversion ratio for the SNPs we discovered was 2.09, as expected from whole-genome sequencing. In comparison, variant calling on the same dataset with the SNPTools pipeline [7] found 72.96 million variants with a slightly lower transition-transversion ratio of 1.98, suggesting a higher number of false positives. Our variant calls were integrated with many other callsets using different variant calling pipelines on the same alignments (including one produced using SNPTools) to produce an integrated callset for Phase 3 of the 1000 Genomes Project. A comprehensive comparison of our callset with other variant callsets on the same data will form part of the 1000 Genomes manuscript.

We evaluated the sensitivity of our callset by comparing to three reference sets:

1. Omni-POLY: Polymorphic sites on the Omni 2.5M genotyping array used by the 1000 Genomes Project.
2. HapMap3-POLY: Polymorphic sites from HapMap Phase 3.
3. 1000 Genomes Phase 1: Variant sites discovered from 1,092 samples in Phase 1 of the 1000 Genomes Project.

Table 1 shows the results of the sensitivity analysis. We can see that our callset has high sensitivity for the Omni-POLY and HapMap3-POLY reference sets. Our callset has relatively low sensitivity for the 1000 Genomes Phase 1 reference set. Further analysis reveals that a majority of the missed variants are singletons from the Phase 1 callset. This behavior is consistent with previous observations of high sensitivity for low frequency variation but reduced sensitivity for singletons and very rare variation when using multisample calling on low-coverage data [9].

**Table 1:** Sensitivity of our callset for standard variant sets.

Reference dataset	Sensitivity (%)
Omni-POLY	94.1
HapMap3-POLY	95.8
1000 Genomes Phase 1	76.5

### Running time and cost

**Fig 2:**
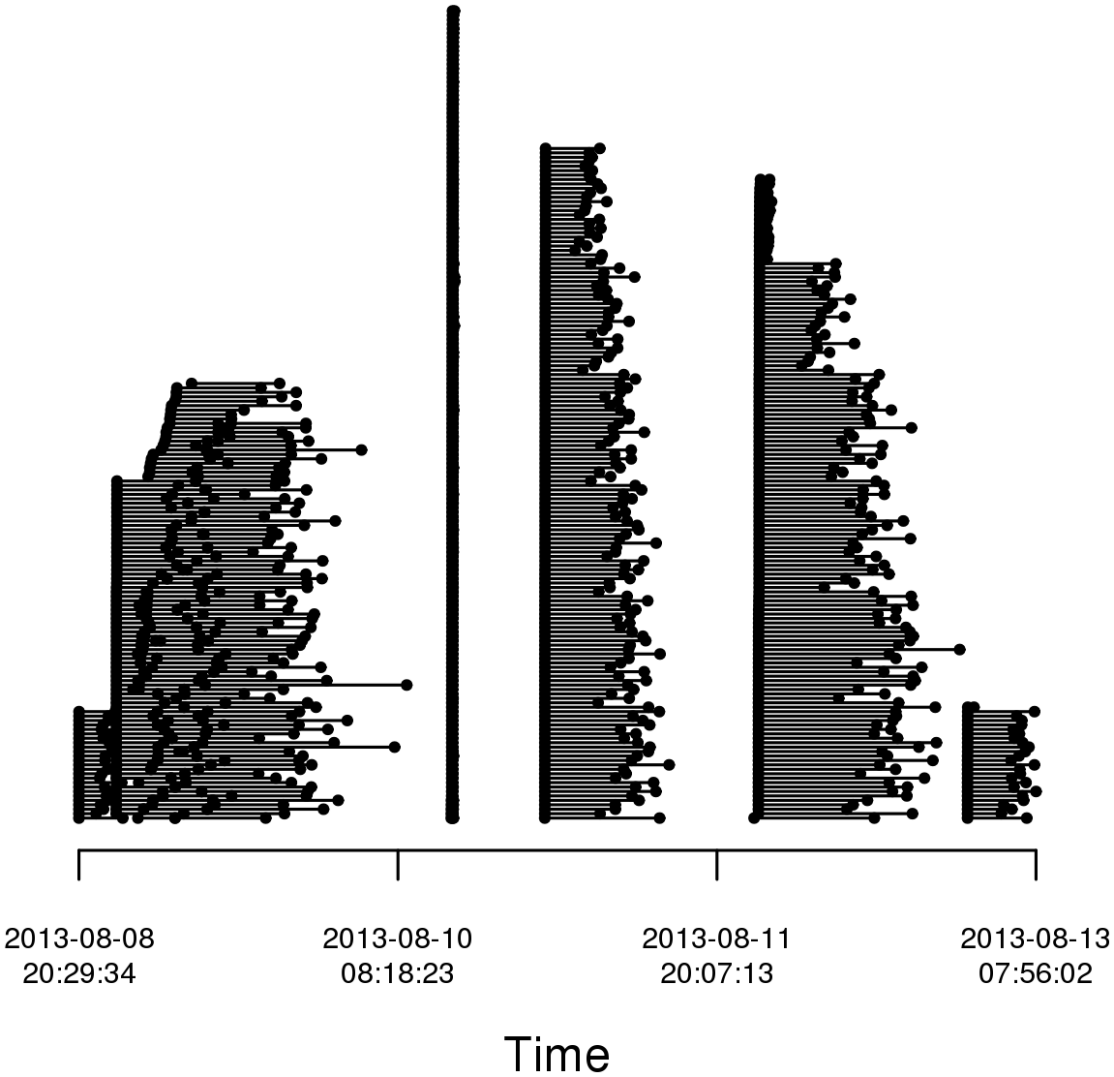
Job timeline. Timeline of jobs run on Amazon EC2. Each job is denoted by a single line segment with start and end times identified by black dots. The very short jobs following “2013-08-10” indicate some jobs that were terminated due to user error in job parameter specifications.

The total computation time for 572 jobs was about 205 days. With parallel execution, these jobs completed in 5 days, representing a 41x speedup. The largest number of concurrent jobs was 223 while the median number of concurrent jobs was 152. The longest job required 22.6 hours of compute time while the median job compute time was 6.3 hours. Fig 2 shows the running time of all jobs plotted as intervals. We submitted jobs in batches (rather than all at once), resulting in the phases of active jobs and gaps between phases. While the EC2 framework allows highly parallel execution, job wait times have been previously seen to increase with submission of thousands of simultaneous jobs requiring large-memory machines [7, 10].

The total cost of all jobs (including failed jobs) was $21,805 while the cost of only successful jobs was $18,590. Based on these costs, we estimate the cost of variant calling per sample to be about $7.33 per sample.

**Fig 3:**
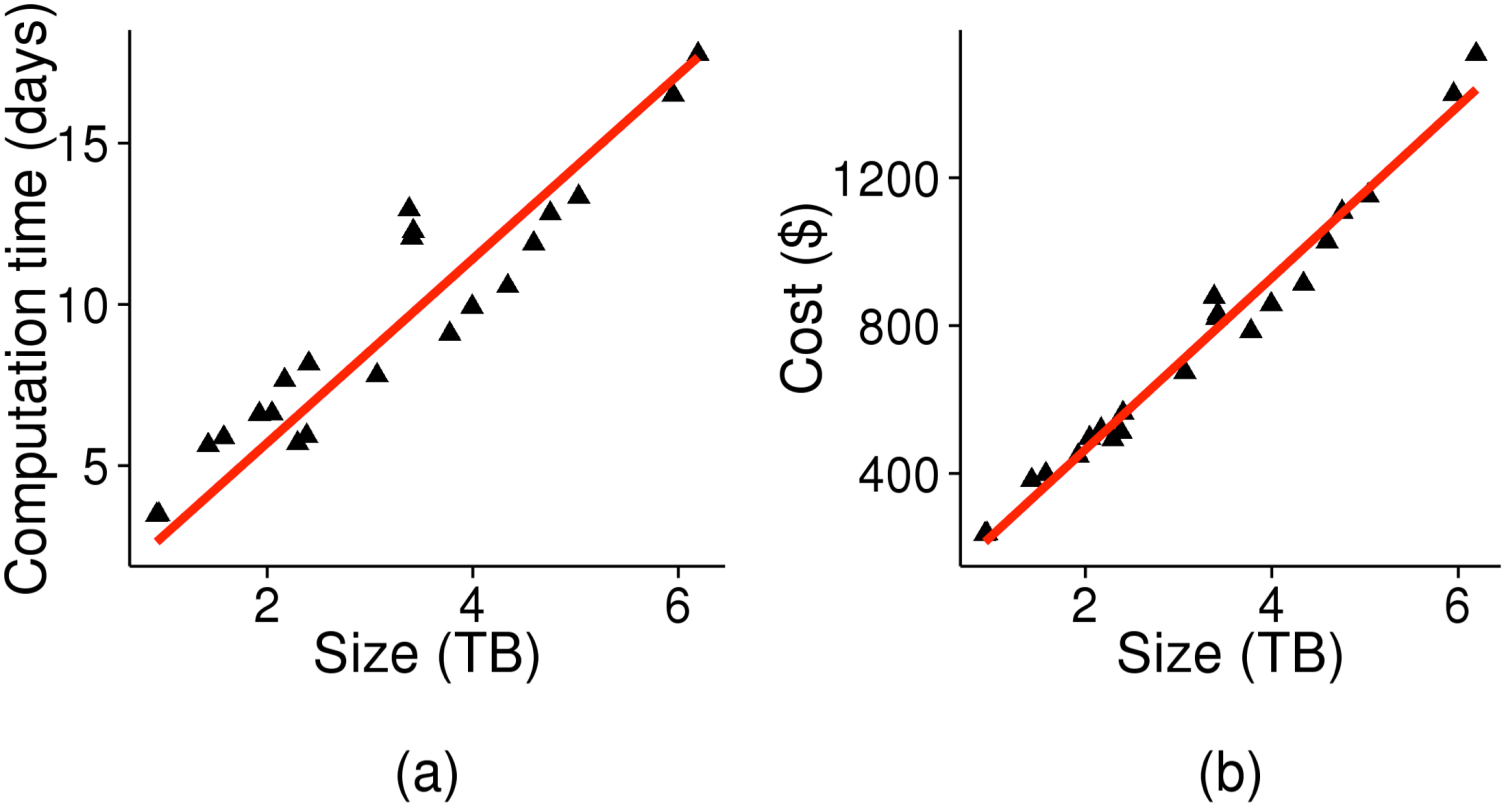
Scalability analysis. (a) Computation time and (b) cost, plotted as a function of alignment size. Each point corresponds to values for one chromosome.

Fig 3 shows how computation time and costs vary with the amount of alignment data. We can see that both computation time and price increase linearly with data size.

## Discussion

Recent studies have sequenced exomes/genomes of hundreds or thousands of individuals. Variant calling on large datasets is challenging due to storage and computing requirements and is rarely reproducible since pipelines are rarely kept intact. Huang et al. [7] estimate that variant calling for 2500 genomes would require 1–2 months of exclusive computing time on a high performance computing cluster (HPCC) with 1000 nodes and 16 TB memory. Few researchers own, or have access to, computing clusters that can facilitate such tasks. Computing clusters are a shared resource at most institutions. Tying up resources for time-consuming variant calling tasks may not be desirable for other cluster users. Furthermore, maintaining computing clusters is expensive. De Alfonso et al. [11] estimate the cost of a 1024-core HPCC to be up to $900,000 per year. An on-demand computing/storage model may thus be attractive to researchers. Cloud computing services provide on-demand computing and storage at low costs. Since workflows can be stored indefinitely in the cloud, an added benefit is the reproducibility of analyses.

We have developed a cloud-based pipeline for variant calling in large samples. It is not constrained by local storage or compute limitations. Using cloud services, we can make the pipeline highly parallel for suitable tasks. With low-cost EC2 instances, we were able to perform variant calling on 2,535 low-coverage human genomes at $7.33 per sample. A direct estimate of the cost of performing the same variant calling analysis on a HPCC is difficult to obtain. Using estimates of HPCC cost [11] and running time [7], we estimate the cost of variant calling on HPCC to be $18,470-$23,610 (see Methods for details). While the cost difference between the cloud-based solution and the HPCC solution may not be significant, the cloud-based pipeline produces results in 5 days compared to 1 month (at least) for the HPCC analysis.

An important consideration for cloud computing applications is the trade-off between running time and cost. An example of this tradeoff can be seen in the deployment of the SNPTools pipeline [7] on the Amazon cloud. Huang et al. [7] report their performance on the same variant calling task that we describe. Table 2 shows a summary of the similarities and differences between the two approaches. The SNPTools pipeline allows users to optimize their setup of EC2 instances and reduce total cost with longer running time. Our pipeline allows users to delegate setup and management of EC2 instances to DNAnexus at higher cost and lower running time (see Methods for details about EC2 instance types).

**Table 2:** Comparing the SNPTools pipeline to our pipeline for the 1000 Genomes Phase 3 variant calling task.

Criterion	Our pipeline	SNPTools pipeline
Variant caller	RTG Population caller	SNPTools
Type of EC2 instances	*spot/reserved/on-demand*	*spot instances*
EC2 instance manager	DNAnexus	Self-managed and optimized
Cost	$18,590	$13,400
Running Time	5 days	11 days

For the low-coverage data of the 1000 Genomes Project, the alignment files for 2,535 genomes had a total size of 70 TB. 30X coverage genomes for the same set of individuals would produce alignment files with a total size of about 300 TB. Transferring and processing a dataset of this size would be time-consuming and computation-intensive. However, there are now solutions available for high-speed transfer of large files, such as GeneTorrent [12] and gridFTP [13]. From Fig 3, we estimate that, for such a dataset, our cloud-based pipeline would need 21 days, assuming a 41x speedup as observed earlier (a computation time of 2.4 years). It would cost $70,000 (≈ $28 per sample) to perform variant calling on this dataset. Such a variant calling task would require 4–8 months of HPCC time on a 1000-node cluster [7] and would therefore be infeasible. On the cloud, it is possible with reasonable cost and running time.

Another advantage of cloud-based analysis is the falling cost of computing and storage. To understand how the cost of analysis would evolve with time, we recalculated the cost of our analysis at the current prices for comparable EC2 instances assuming that the run-time would be identical. In practice, this estimate will be conservative since the faster processor and solid state drives should allow faster completion. At current prices, our analysis would have cost $6.19 per sample ($15698.73 total), representing a 15% reduction when compared to our cost of $7.33 per sample ($18,590 total).

Recently, cloud-based pipelines have been used to analyze thousands of samples efficiently. Reid et al. [6] describe the Mercury pipeline for variant calling and annotation. They use the Atlas2 variant caller [14] and DNAnexus to analyze over 10,000 genomes and exomes. The variant calling tasks they describe (singlesample calling) are different from ours (multisample calling) in memory and computing requirements. But their study demonstrates that cloud-based variant calling can be applied to large numbers of genomes.

An important feature of cloud-based pipelines is the ease of reproducibility. With large amounts of sequencing data being generated and analyzed rapidly, reproducibility is an important requirement for variant calling. Since cloud-based analyses must necessarily be run on multiple computers, which may or may not have the necessary software programs installed already, analysis pipelines often include copies of not only workflows but also software and virtual machines. For instance, our variant calling pipeline on DNAnexus archives the script used, a copy of the version of the RTG caller used and even versions of auxiliary software such as the *java* environment required for the analysis. This allows analyses to be re-run easily using the same versions of software programs to verify results.

Cloud-based systems may not be the optimal solution for certain scenarios, depending on desired scalability, cost constraints, need for always-available resources. etc. Studies of the total cost of ownership, response time, resource usage, etc. [10, 15–17] provide a comprehensive comparison of cloud-based systems and HPCC frameworks. A challenge in the use of cloud-based systems is the setup of computing instances and storage for analysis tasks. Services like DNAnexus and Galaxy Cloudman [18] provide accessible interfaces to set up and manage cloud resources. This is helpful to new users who may not be familiar with efficient cloud computing resource management. A drawback of this approach is a limited ability to fine-tune optimization of resources. Genomic data security and privacy are concerns that are being addressed by the community through development of compliance standards for service providers. Another challenge for the use of cloud-based analysis pipelines is the requirement for data transfer to the cloud for storage and analysis. Network speeds can be a bottleneck if users need to upload large amounts of data to the cloud for analysis. As sequencing projects grow, local storage will become difficult and cloud storage will become a primary storage mechanism. This will remove the need for time-consuming data transfer and make cloud-based analyses popular in the future.

## Methods

For our analysis of low-coverage sequence data from the 1000 Genomes Project, we used the multisample population caller from Real Time Genomics, since multisample calling has been shown to improve rates of variant discovery in low-coverage data [1]. Deploying pipelines to the Amazon cloud can be difficult for new users, especially if the goal is to maximize parallelism at minimal cost. To avoid these difficulties and deploy the Real Time Genomics variant caller on the Amazon cloud, we used the DNAnexus service. We describe all these components below in more detail.

### Real Time Genomics population caller

**Fig 4:**
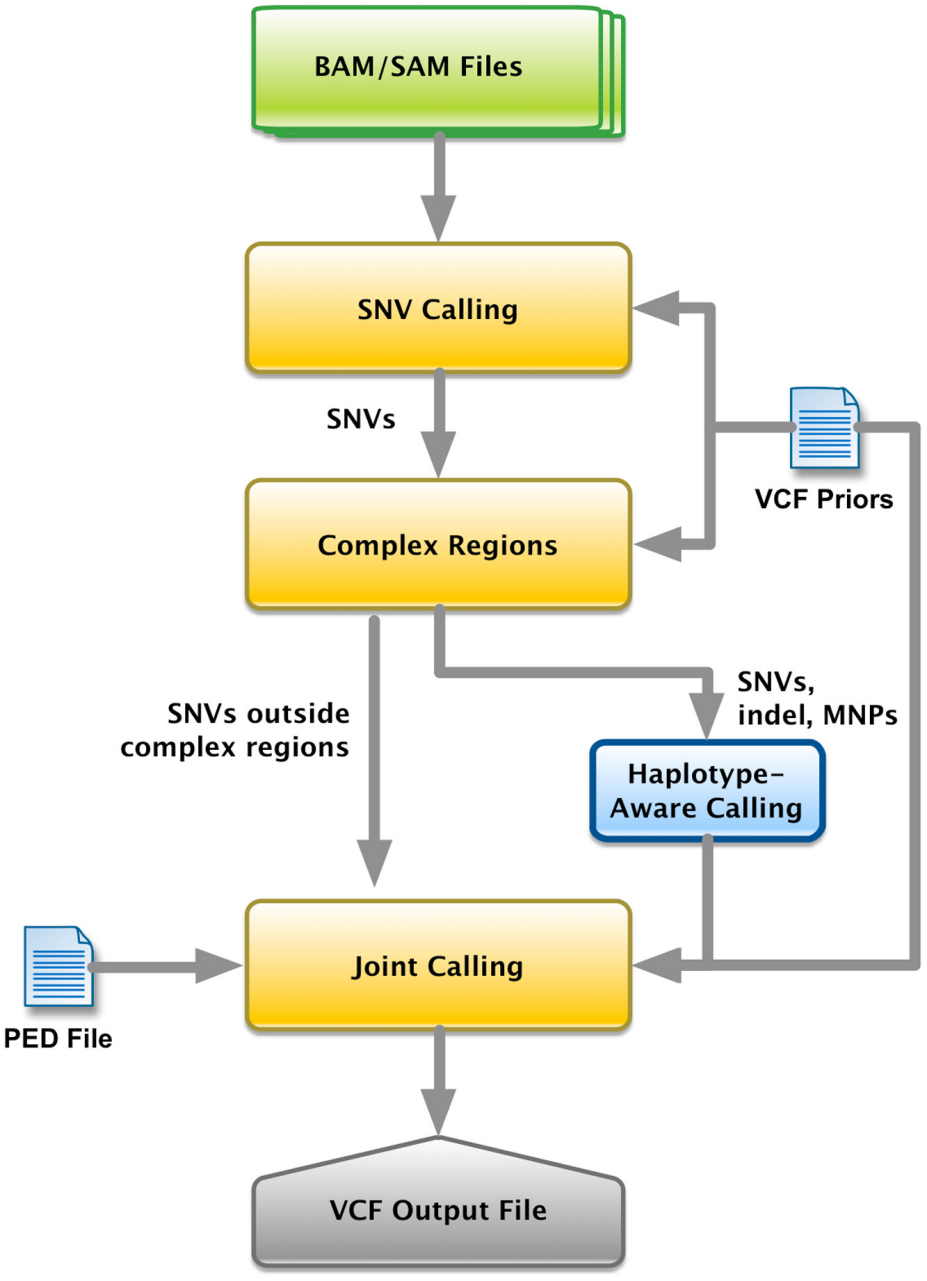
RTG population caller workflow. Diagram showing the workflow of the RTG population caller.

The Real Time Genomics (RTG) population caller uses a Bayesian framework to iteratively update site-specific priors until convergence based on the calls of the complete sample at each step. This framework is useful for low-coverage data, such as the 1000 Genomes data, since variants may be missed in some individuals due to insufficient read support. Fig 4 shows a flowchart describing the algorithm used by the RTG population caller. Cleary et al. [8] describe the method and underlying algorithms in more detail. For our analysis, we used RTG Core version 3.1.2.

### Amazon cloud

Amazon Simple Storage Service (S3) provides cheap storage in the cloud. Data stored in the Amazon cloud can also be downloaded quickly using many simultaneous network connections. Even though sequencing data from the 1000 Genomes Project has low sequencing depth (8–10x), the large number of samples has resulted in alignment files with a total size of ∼70 TB. These alignments are hosted on Amazon S3 for convenient access (URL: http://s3.amazonaws.com/1000genomes/). We used the http retrieval mode of *samtools* [19] to download BAMs for our analysis.

For computing, we used the Amazon Elastic Compute Cloud (EC2) system. This gives users access to a variety of machines with different processing and memory capabilities. For variant calling, we *m2.4xlarge* instances (64-bit 8 CPU machines with 68.4 GB memory).

### DNAnexus

The Amazon EC2 system allows users to either reserve computing instances (*reserved instances*), request computing resources on demand (*on-demand instances*) or bid for spare computing instances (*spot instances*) at variable cost. *Reserved instances* are always available but are expensive. *On-demand instances* provide uninterrupted service but may not always be available. Bidding for *spot instances* allows users to reduce costs. However, these instances are recommended only for time-flexible and fault-tolerant tasks. To avoid the problem of managing these instance types while optimizing cost and running time, we used the DNAnexus service, which automates the management of EC2 instances and provides a convenient interface with the Amazon cloud system. We were thus able to use EC2 instances at low cost without having to manage their allocation or availability. Data transfer can be a bottleneck in large variant calling tasks. Using the Amazon EC2 computing system with the alignment data stored in Amazon S3 allowed us to perform fast parallelized data transfer.

### Splitting data for parallelism

For parallelism, we performed variant calling separately on each chromosome. The RTG population caller implicitly assumes Hardy-Weinberg equilibrium information during the estimation of the site-specific priors in the sample. Population substructure can lead to artifacts such as reduced heterozygosity in Hardy-Weinberg calculations. We therefore separated variant calling across populations as well. For the 26 populations of 1000 Genomes Phase 3, this created 572 independent jobs (26 populations × 22 chromosomes).

### Cost estimate for HPCC analysis

De Alfonso et al. [11] estimate the cost of a 1024-core HPCC to be $160,000 (assuming 0% usage)-$900,000 (assuming 100% usage) per year. Huang et al. [7] estimate the variant calling task to require 1–2 months at 100% usage on a HPCC of this size. Therefore, the period of variant calling would have a cluster cost of $75,000-$150,000. However, for such a cluster, this cost can be amortized over the entire year. Assuming the HPCC to be at 100% usage during variant calling (as per [7]) and 0% usage otherwise, the amortized cost of variant calling will be $18,470 if it requires a 1 month period and $23,610 if it requires 2 months.

### Software

The source code for our DNAnexus application is available for download in the Supporting Information. Due to licensing restrictions, we are unable to include the RTG variant calling code in the released code. Interested researchers can email info@realtimegenomics.com for free academic licenses.

## Supporting Information

S1 File Source code for our pipeline. (TAR)
